# The novel arthroscopic subscapular quadriceps tendon–bone sling procedure provides increased stability in shoulder cadavers with severe glenoid bone loss

**DOI:** 10.1007/s00167-020-05900-1

**Published:** 2020-02-14

**Authors:** Jan Arild Klungsøyr, Terje Vagstad, Manuel Ferle, Jon Olav Drogset, Solveig Roth Hoff, Andreas F. Dalen, Christof Hurschler, Christian von Falck, Peter Klungsøyr

**Affiliations:** 1 Orthopedic Department, Ålesund Hospital, Møre and Romsdal Health Trust, Ålesund, Norway; 2 Faculty of Medicine and Health Sciences, Norwegian University of Science and Technology, Trondheim, Norway; 3 Department of Radiology, Møre and Romsdal Hospital Trust, Ålesund, Norway; 4 Department of Circulation and Medical Imaging, Faculty of Medicine and Health Sciences, NTNU, Trondheim, Norway; 5 Labor für Biomechanik und Biomaterialien, Orthopädische Klinik der Medizinischen Hochschule Hannover-Annastift, Hannover, Germany; 6 Institut für Diagnostische und Interventionelle Radiologie Medizinische Hochschule Hannover (MHH) Hannover, Hannover, Germany; 7 Norwegian University of Science and Technology, Trondheim, Norway; 8 Trondheim University Hospital, Trondheim, Norway

**Keywords:** Shoulder instability, Arthroscopic sling procedure, Quadriceps tendon bone graft, Subscapular tendon, Biomechanical cadaver study, Glenoid bone loss

## Abstract

**Purpose:**

Treatment of anterior glenoid bone loss in patients with recurrent anterior shoulder instability is a challenge. The subscapular sling method with quadriceps tendon bone (QTB) graft is a modification of the subscapular sling with a semitendinosus (ST) graft. The aim of the study was to test the biomechanical stability of the QTB sling procedure in human shoulder cadavers with severe anterior glenoid bone loss.

**Methods:**

Fourteen cadaveric shoulders were tested with a force–moment-guided robot in three conditions: physiologically intact, anterior glenoid bone resection, and the subscapular sling procedure with a QTB graft. Joint stability was measured in anterior, anterior inferior and inferior directions in four glenohumeral joint positions: 0° and 60° of glenohumeral abduction, with each at 0° and 60° of external rotation. Maximum external rotation was measured at 0° and 60° glenohumeral abduction. Computer tomography scans were obtained preoperatively to plan the glenoid bone resection, as well as postoperatively to calculate the proportion of the glenoid bone actually resected.

**Results:**

Significantly decreased translations were observed in the shoulders with the QTB sling compared to the intact joint and the glenoid bone loss model. No significant differences in maximum external rotation were observed between the three different conditions.

**Conclusion:**

This biomechanical study revealed a significant stabilizing effect of the arthroscopic subscapular QTB graft sling procedure in human shoulder cadavers without compromising external rotation. Clinical trials may reveal the usefulness of this experimental method.

## Introduction

Patients suffering from an anterior shoulder dislocation are restricted in their activities of daily living. The existing methods for surgical stabilization of a shoulder joint with glenoid bone loss render good results, but have complications and recurrent dislocations [3, 4, 30]. Bone grafting procedures as for instance the Latarjet can be performed after failures of former interventions, but also in cases with insufficient anterior structures with or without significant anterior glenoid bone loss [1, 2, 22, 27]. Despite the fact that the Latarjet procedure has been broadly applied [30] and is performed both open and arthroscopically [9, 16], complication rates are quite high (15–30%) [6, 12, 13, 16]. A systematic review [12] reported up to 30% complication rate of the Latarjet procedure with loss of motion, pain, graft non-union, nerve injury and secondary osteoarthritic changes as the most common complications. In cases of failure, a revision is difficult because of the distorted anatomy, particularly with regards to the musculocutaneous nerve, which is displaced together with the conjoined tendon. The interaction between the conjoined tendon and the subscapular tendon in the Latarjet procedure results in a sling-type structure. The importance of this sling phenomenon has been well described in cadaveric studies [11, 40, 43]. The subscapular sling procedure with semitendinosus (ST) graft [20] has recently been developed, whereby a sling around the upper part of the subscapular tendon is constructed using a ST graft. As a result, this procedure does not alter the anatomy to as large a degree as the Latarjet procedure [20]. When performing the ST sling procedure, all the instrument handling takes place lateral to the conjoined tendon, which potentially reduces the risk of harm to nerves and vessels. As a consequence of the previous convincing biomechanical investigations of this new method [37], we decided to further explore the potential of an arthroscopic subscapular sling procedure which utilizes a quadriceps tendon bone (QTB) graft. The aim of this study was to biomechanically evaluate this new stabilizing technique on human cadaveric shoulders in a glenoid bone loss model.

The hypothesis was that the arthroscopic subscapular sling procedure with QTB graft would grant stability to a glenohumeral joint in an anterior glenoid bone loss model without reducing the maximum external rotation. The development of new arthroscopic surgical techniques with less potential complications may enhance the treatment options available for patients suffering from shoulder instability.

## Materials and methods

This study was approved by the local ethical committee at the Hannover Medical School (Number 3005-2016).

Sixteen fresh frozen human cadaveric shoulders were acquired (Science Care, USA). The cadaveric shoulders obtained showed no evidence of radiological osteoarthritis (OA) and did not have any medical history of shoulder treatment, anterior shoulder instability or injury. Two specimens were not included in the analysis due to fracture of the scapula during experimental surgery in one case, and an occult fracture of the coracoid in another. The remaining 14 cadaveric shoulders were included in the study (6 female, 8 male, mean age 56.7, SD 5.9 years). All specimens were stored at − 23 °C and were thawed over a period of at least 12 h before testing. The shoulder specimens included the intact glenohumeral joint with the scapula, clavicle and humeral shaft with muscles, subcutaneous tissue and skin.

### Quadriceps tendon–bone grafts

Quadriceps tendon–bone (QTB) grafts were taken from cadaveric human knees (Science Care, USA) without any former known injury or disease. The minimum attainable tendon length was found to be 8 cm. The bone graft was 2.5 cm long and had a cross section of approximately 10 × 10 mm. The cut of the bone block side placed to face the glenoid rim was made with a small angulation to prevent the block having a smaller radius than the specimen glenoid and thus resulting in a bone block flush with the physiologic surface of the glenoid. The cranial end of the bone block was recessed to prevent injury of the subscapular tendon.

### CT scanning

All scans were acquired on a 64-slice multidetector row CT (VCT, GE Healthcare, Chalfont St. Giles, UK) using the following parameters: slice thickness: 0.625 mm; reconstruction increment: 0.4 mm; tube voltage: 120 kV; tube current–exposure time product: 365 mAs (fixed); pitch: 0.5312; rotation time: 0.8 s. Images were reconstructed using a sharp kernel (‘bone plus’, FOV: 350 mm). The resulting images were exported in the DICOM file format for further analysis.

### Creation of severe anterior glenoid bone loss

**Fig. 1:**
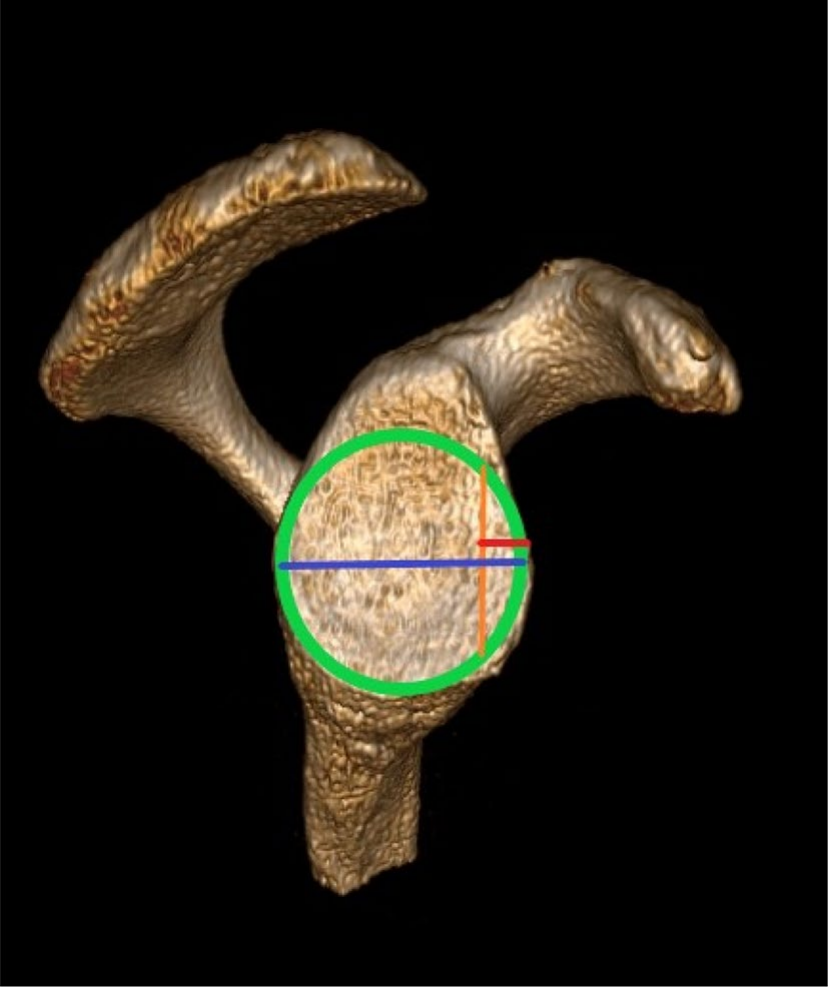
Preoperative planning image. Glenoid width (blue), diameter of best-fit circle (green), 20% calculated glenoid bone defect (red/orange)

**Fig. 2:**
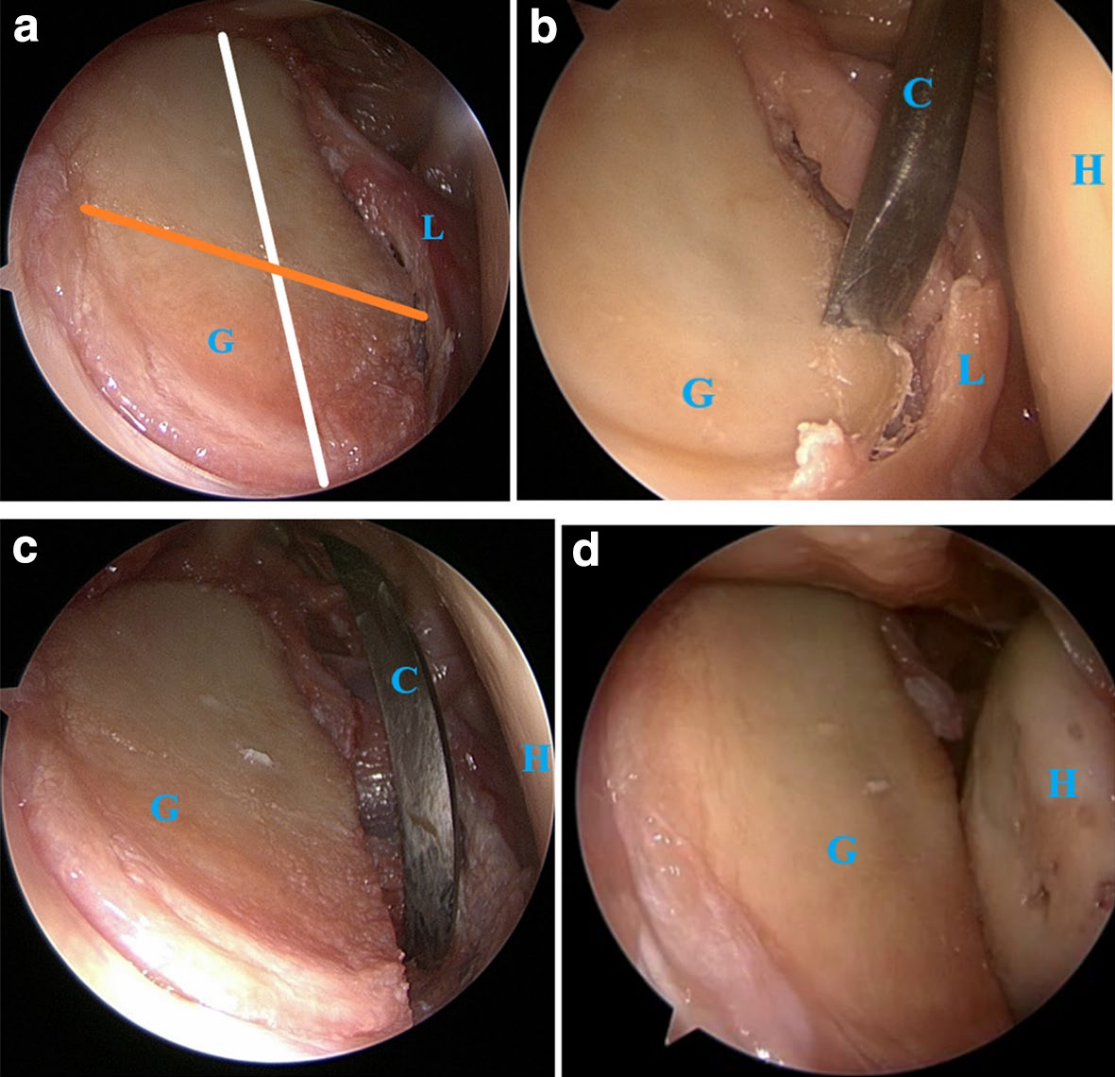
**a** The resection of the anterior glenoid was performed parallel to the white line between 12 and 6 o’clock, orange line marks the diameter of best-fit circle. **b** The customized chisel. **c** After resection of the calculated 20% glenoid bone loss. **d** Anterior dislocation of the humeral head in the lesion condition. Chisel (C), glenoid (G), humeral head (H), labrum (L)

The recommended preoperative planning imaging of the glenoid is a three-dimensional computed tomography after subtraction of the humeral head [35]. The glenoid bone loss can be quantified as a ratio of the defect width against the diameter of the displayed perfect circle of the inferior two-thirds of the glenoid [17, 35]. A 3D CT was performed of all shoulder cadavers. A perfect circle was drawn in the lower two-thirds of the glenoid on a sagittal cut of the glenoid face using the quantifying method described (Fig. 1) [35]. The diameter of the circle was measured and 20% of this diameter taken as the width of anterior glenoid bone fragment to be resected. The labrum was detached and removed from 2 to 6 o’clock. The resection of the anterior glenoid was performed along a line parallel to the axis between the 6 and 12 o’clock positions similar to the clinical appearance of a glenoid bone loss condition (0° osteotomy model) (Fig. 2) [29]. Chisels with width between 3 and 7 mm were used to define the exact resection line on the anterior rim of the glenoid. Postoperative CT scans were used to measure the remaining glenoid width and to verify the degree of glenoid resection actually achieved.

### Subscapular sling procedure with a quadriceps tendon bone (QTB) graft

**Fig. 3:**
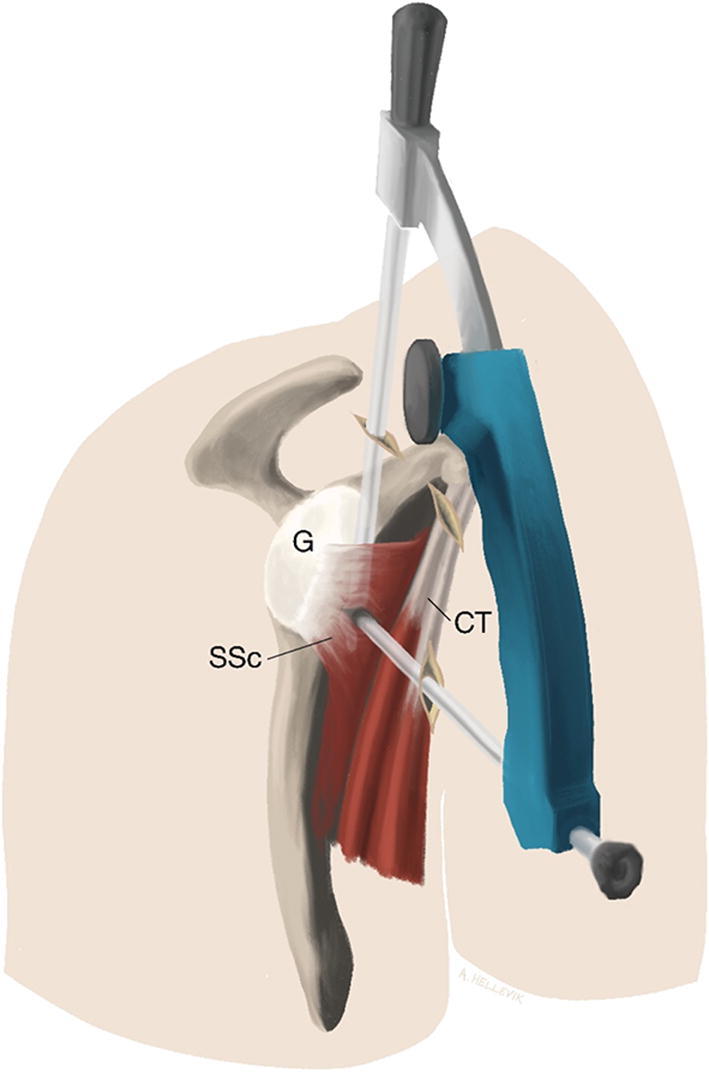
Creating the slit in the subscapular tendon. Subscapular tendon (SSc), conjoined tendon (CT), glenoid (G). The lower anterior portal is created lateral to the conjoined tendon to avoid structures at risk (axillary and musculocutaneous nerves) using an aiming device for AC-joint repair (Arthrex GmbH), switching stick and halfpipe instruments. With help of a switching stick and dilatators, the slit is expanded and a halfpipe is introduced through the lower anterior portal. A banana knife was used to enlarge the slit from medial to lateral

**Fig. 4:**
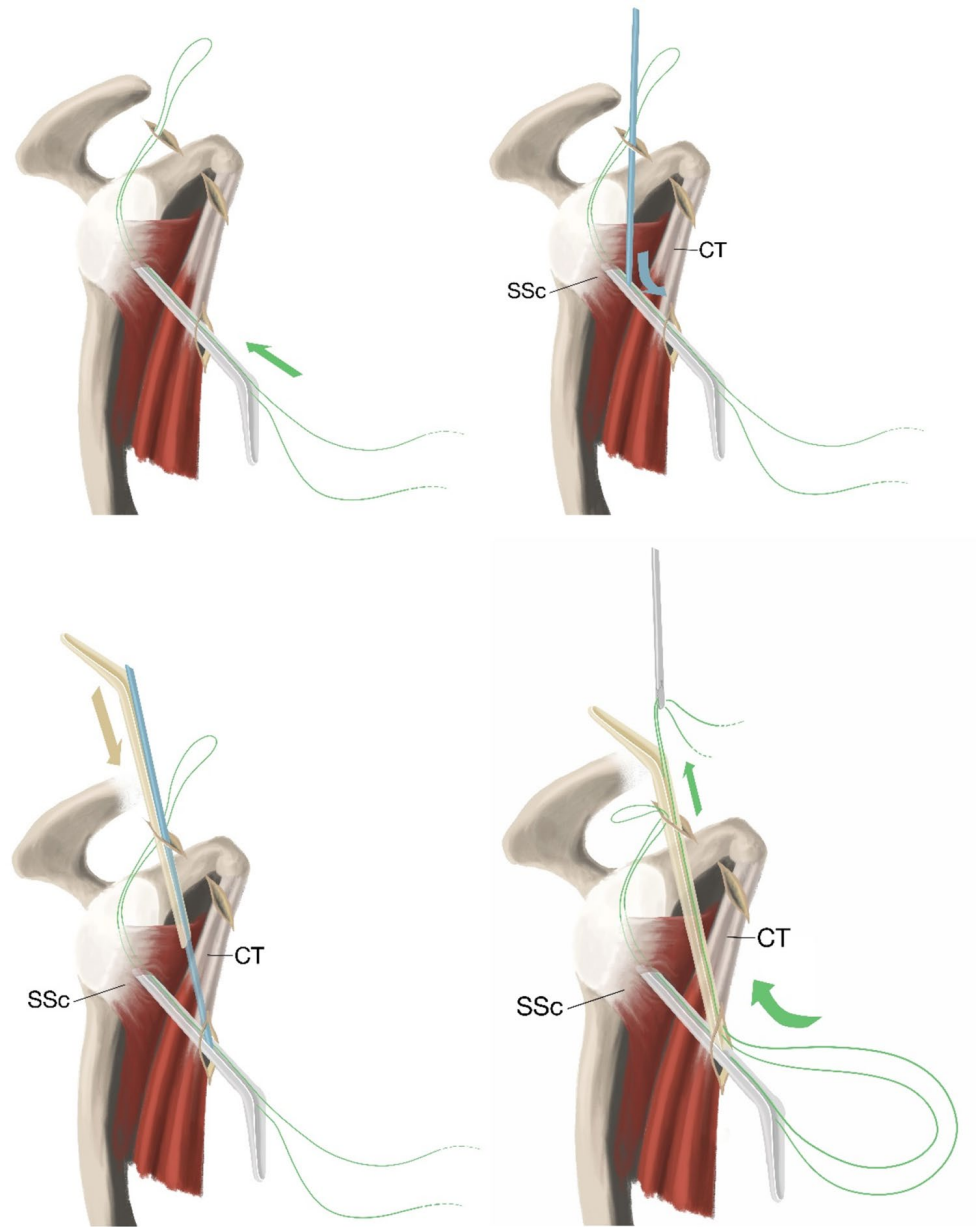
A passing suture is introduced into the joint through the lower anterior portal over the halfpipe and retrieved into the anterior superior portal. A switching stick is used to clear the layer anterior to the subscapular tendon and advanced carefully into the halfpipe situated in the lower anterior portal. A second halfpipe is introduced and a grasper pulls the passing suture out of the anterior superior portal around the subscapularis. Subscapularis (SSc), conjoined tendon (CT)

**Fig. 5:**
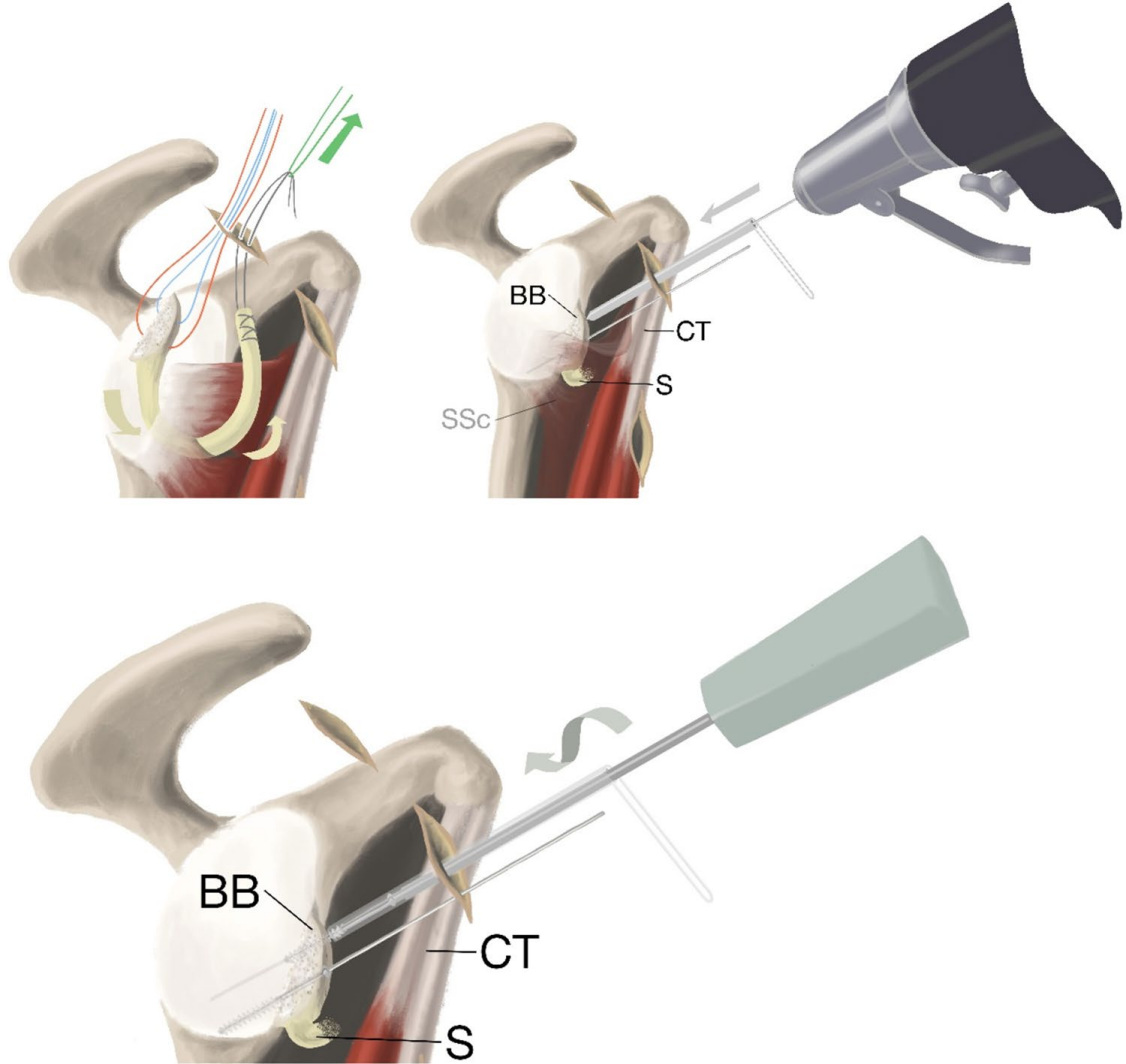
Green passing suture used to pull the QTB graft into the joint. Color-code sutures aid placement of bone block on glenoid with K-wires and finally fixation with screws. Bone block (BB), sling (S), conjoined tendon (CT) and subscapular tendon (SSc)

**Fig. 6:**
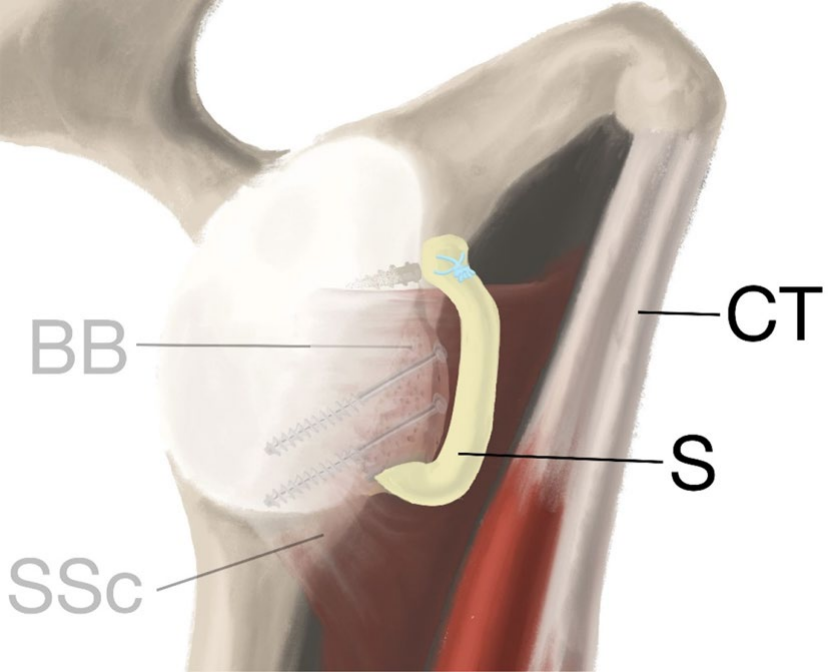
The subscapular quadriceps tendon–bone sling. Bone block (BB), sling (S), conjoined tendon (CT) and subscapularis (SSc)

The diagnostic arthroscopy and the following procedures were performed “dry” (i.e. in air instead of water). Standard posterior, anterior, and anterosuperior portals were placed with the anterior portal created cranial to the superior edge of the subscapularis tendon. The subscapularis tendon was cleared in the front. The axillary nerve was identified. All instrumentation was performed lateral to the conjoined tendon, thus preserving the musculocutaneous nerve. The lower anterior portal was created lateral of the conjoined tendon from anterior to posterior direction using aiming guide, switching stick and halfpipe instrument (Fig. 3). A slit was made in the longitudinal direction between the lower and middle third of the subscapular tendon. This was achieved with 20° of external rotation of the shoulder and under visual control cutting from medial to lateral. Two holes were drilled in the block using 0.62-mm drill guides prior to introducing the graft into the joint. Two suture threads with color codes were pulled through the holes in the block to facilitate placement of the block and drilling into the anterior glenoid rim. Stiches were placed in the proximal quadriceps tendon graft end for ease of handling. A suture was introduced from the anterosuperior portal around the subscapular tendon back out of the anterosuperior portal (Fig. 4). The tendinous graft end was connected to this suture and pulled into the joint and around the subscapular tendon through the anterosuperior portal. A K-wire was introduced in the cranial hole in the block and together with the color-coded sutures used to achieve the right placement of the bone block on the anterior rim of the glenoid. A second K-wire drilled through the anterior portal was placed to achieve rotational stability of the block (Fig. 5). After fixation of the bone block with two cannulated partially threaded 3.75-mm screws (Arthrex GmbH), the tendinous part of the graft was retrieved into the joint. A Corkscrew 4,5-mm anchor (Arthrex GmbH) was placed at the cranial end of the bone block into the glenoid rim and the graft pulled into the rim while tensioning and pulling the attached sutures (Fig. 6).

### Preparation and mounting of the shoulder cadavers

Neutral humeral internal–external rotation was defined by flexing the elbow to 90° and inserting a K-wire into the humeral shaft from anterior to posterior approximately 16 cm below the top of the humeral head and parallel to the forearm. Skin, soft tissue and muscles were resected below the K-wire. The humerus was osteotomized 5 cm below the K-wire and embedded in a brass cylinder for mounting using a two-component polyurethal casting resin (Rencast FC 52/53 Isocyanate, Polyol FC 53, Filler DT 082; Huntsman Corp.; The Woodlands; USA). The skin, soft tissue and muscles of the distal two-thirds of the scapula were resected and scapula potted using the same resin molded in a custom-made box. The scapula block was then secured to a mounting plate using three threaded rods and tilted 10° forward of the scapula to imitate the physiologic tilting of the scapula on the thorax. The rigid attachment allowed stable mounting of the specimens during surgery and robot testing. All shoulders were vented by injection of 20 ml of ambient air using a small needle to prevent bias from the existing negative pressure in the joint [38].

### Test setup and protocol

The biomechanical tests were performed using a robot-assisted shoulder simulator as used in several previous studies [39–42].The setup consisted of a scapula mount and an industrial robot (KR16-2, KUKA AG, Augsburg Germany) which was equipped with a six-component force–moment sensor (Delta, ATI Industrial Automation, Apex, USA) to which the humerus was attached. While the mounting tower firmly held the scapula, the robot setup was able to apply force- and moment-controlled motions to the glenohumeral joint. The setup allowed motion control with a repeatability of 0.04 mm and measurement of forces and moments with a resolution of less than 0.25 N and 7.5·10^−3^ Nm. For implementing the robot control, a global coordinate system, which is fixed in space, was defined as follows: The *x*-axis was directed medially parallel to the previously defined scapula plane. The *y*-axis was defined as being perpendicular to the scapular plane and directed posteriorly. And finally, the *z*-axis was defined by the *x*- and *y*-axis and directed superiorly. Furthermore, a humeral coordinate system was defined at the geometric center of the humeral head to describe the motion of the humerus with respect to the scapula, as previously described [39, 42]. The humerus coordinate system was defined to be co-directional to the global coordinate system after centering the humeral head in the glenoid with the arm hanging under its own weight in neutral rotation. An Euler angle system was used to describe humeral rotation with respect to the scapula: rotation about the *x*-axis (flexion and extension); rotation about the *y*-axis (abduction in the scapular plane); rotation about the long axis of the humerus (internal–external rotation).

Translational joint stability was defined as the anterior, anteroinferior, and inferior translation occurring under a load of 30 N in each respective direction while simultaneously centering the humerus in the glenoid with a 30 N medially oriented force. The joint was allowed to translate freely in anterior–posterior, superior–inferior, and medial–lateral directions, while all rotations were held constant. The maximum external rotation was defined as the maximum angle that could be reached by applying an external rotation moment of 2 Nm, while simultaneously applying a medially oriented centering force of 30 N. During external rotation, the joint was again allowed to translate freely in anterior–posterior, superior–inferior and medial–lateral directions, while the abduction and flexion angle were held constant.

All shoulders were tested sequentially under three conditions: ventilated but otherwise intact joint, with the 20% glenoid bone resection, and finally after the completed subscapular sling procedure with a quadriceps tendon–bone graft. Glenohumeral translation was performed and measured in mm (to one decimal place) for all three conditions in four positions: at 0° and 60° of glenohumeral abduction and 0° and 60° of external rotation, respectively. The measurement of the translations was done by recording the end-effector movements of the robot. These translations were acquired by the sensors integrated in the robot system. Maximum external rotation was tested in 0° and 60° of glenohumeral abduction. The 60/60 position of glenohumeral movement equals the combined glenohumeral–scapulothoracic 90/90 position where an anterior shoulder dislocation usually occurs in a clinical setting. After the completed biomechanical testing, the cadavers were arthroscopically inspected before refreezing. Postoperative CT scans were subsequently performed to measure the remaining glenoid width and calculate the actual amount of anterior glenoid bone resected. The position of the bone graft used for the subscapular QTB sling procedure was also determined.

### Statistical analysis

Measured translations and rotations were compared between the intact joint, anterior glenoid bone loss of 20% and the sling procedure at each tested position using analysis of variance (ANOVA) with the specimen as the repeated measure followed by a Tukey post hoc test. The level of significance was set at *α* = 0.05. Statistical analyses were carried out using the R software package (version 3.3.2; R Foundation for Statistical Computing). The sample size was calculated based on data from the previous study of the subscapular sling [37] by use of the software G Power and Stata. The results were: with a mean of 15.9 mm (SD 8.1) of translation (anterior direction) in the lesion condition, and 7.5 mm (SD 3.8) in the sling condition, with a 95% CI and a power of 90, the required sample size (*N*) is 11.

## Results

### Translation and external rotation

Mean glenohumeral translations of the three conditions (intact, bone lesion and sling) measured in 0° and 60° of glenohumeral abduction, with each at 0° and 60° of external rotation are displayed in Table 1.

**Table 1:** Translation in mm in different positions and directions, standard deviation in brackets

Abduction/rotation	Direction	Intact (SD)	Bone lesion (SD)	Sling (SD)	*P* value
0°/0°	Anterior	19.1 (7.0)	25.3 (6.3)	7.3 (4.8)	< 0.001*
	Inferior	9.9 (5.4)	13.9 (6.8)	12.4 (7.5)	< 0.03***
	Anterior inferior	19.8 (8.5)	27.2 (7.9)	7.5 (5.6)	< 0.001*
0°/60°	Anterior	12.0 (6.3)	17.8 (8.4)	5.0 (3.4)	< 0.001 *
	Inferior	8.0 (6.2)	14.4 (7.3)	9.3 (8.0)	< 0.02 **
	Anterior inferior	14.0 (7.4)	21.5 (8.0)	5.0 (6.3)	< 0.001*
60°/0°	Anterior	15.9 (7.5)	20.4 (10.0)	7.2 (4.1)	< 0.001*
	Inferior	6.0 (3.5)	14.6 (9.8)	11.2 (6.8)	< 0.001 *
	Anterior inferior	15.7 (7.3)	24.3 (12.2)	7.8 (5.4)	< 0.05*
60°/60°	Anterior	13.3 (5.8)	17.7 (11.6)	10.0 (11.1)	< 0.01**
	Inferior	11.2 (5.3)	11.1 (7.1)	16.2 (7.1)	< 0.001*
	Anterior inferior	13.3 (5.9)	17.0 (11.2)	6.8 (5.8)	< 0.001**

Significant differences are marked in the right column with an Asterix (sling vs lesion and intact (*), sling vs bone lesion (**) sling vs intact (***))

No significant differences in external rotation were found between the three different conditions (Table 2).

**Table 2:** External rotation in degrees in 0° and 60° of abduction, standard deviation in brackets

Abduction	Intact ° (SD)	Bone lesion ° (SD)	Sling ° (SD)	*P* value
0°	87.9 (22.9)	95.3 (20.3)	90.3 (31.0)	n.s
60°	91.0 (39.5)	90.2 (43.8)	85.1 (43.1)	n.s

### Glenoid measurement and resection

The preoperative CT scans revealed a difference in glenoid sizes in the investigated specimens. The mean diameter (anteroposterior width in the best-fit circle) of the glenoid was 28.3 mm (range 22.6 to 33.4 mm, SD 3.4). The width of the resected bone segment to obtain a 20% glenoid bone loss ranged preoperatively from 4.5 to 6.7 mm with a mean of 5.7 mm (SD 0.7). Postoperative CT scans revealed that the mean remaining glenoid width was 23.3 mm (range 18.0 mm to 28.2 mm SD 2.9) The mean width of the resected glenoid bone was 5.1 mm (equals 18% glenoid bone loss) (range 3.6 to 6.2 mm, SD 0.8). There is a significant difference between the means of the calculated (target) and actually resected glenoid bone fragments (*p* = 0.006). All cadaveric shoulders dislocated anteriorly with manual external rotation and abduction after the glenoid resection was performed.

### Integrity of bone block after robot testing

Postoperative arthroscopic inspection after the robot testing revealed no visible changes of the screws and no displacement of the bone block. However, CT scans postoperatively revealed three specimens with changed positions of the screws and fractured bone blocks. The CT scans showed that all bone grafts were placed within the radius of the lower circle of the glenoid and that the cranial end of the bone block was placed below the 2 o’clock position in all cadavers and covered the resected glenoid area.

### Problems encountered during surgery

Some challenges occurred during the arthroscopic surgery. In one cadaver, the proximal tendon graft fixation loosened while testing the shoulder manually under arthroscopic visualization. This was resolved by applying a new anchor and refixation of the graft before the robot testing was completed. One of the bone blocks fractured at the insertion point of the tendon and the graft had to be replaced. To minimize the risk of fracture or rupture of the tendon at the insertion point, the inferior screw was, therefore, placed as close as possible to the superior screw.

## Discussion

**Fig. 7:**
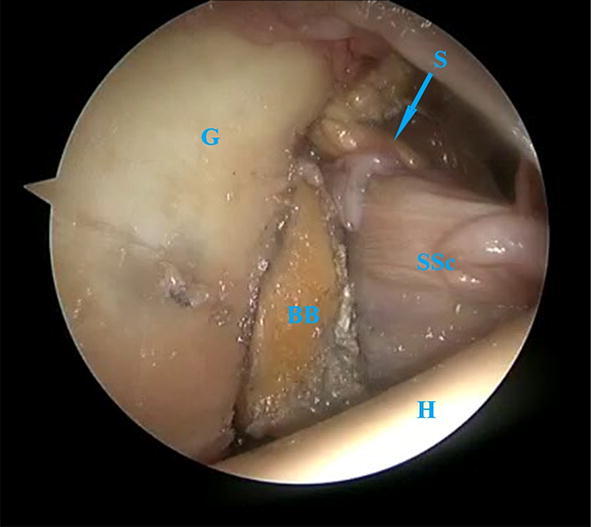
The completed sling in an arthroscopic view. Subscapular tendon (SSc), bone block (BB), glenoid (G), humeral head (H), sling (S)

The most important finding in this study is the decreased translation and thus increased stability in the cadaveric shoulders with the completed sling compared with the intact joint and glenoid bone loss condition. None of the shoulders dislocated manually and the translations were significantly decreased in anterior and anterior-inferior direction compared with the intact joint and the 20% anterior glenoid bone loss status. There was no difference between the conditions regarding the range of external rotation motion. The arthroscopic subscapular sling procedure stabilizes the shoulder by means of a QTB graft placed on the glenoid and around the subscapular tendon (Fig. 7). The aim is to restore the anterior glenoid bone loss and subsequently provide dynamic and static stability to prevent anterior translation and dislocation of the humeral head.

There is no general agreement regarding the definite percentage of glenoid bone loss that indicates the need for a bone grafting procedure to achieve glenohumeral joint stability [26]. Some authors recommend a bony procedure such as the Latarjet for patients with glenoid bone loss greater than 20% of the glenoid width [4, 13, 44]. Burkhart et al. reported that an anteroposterior glenoid defect greater than 25% would need a bone grafting procedure and concluded that a Bankart repair alone would not give sufficient stability in such cases [4]. Other studies have reported that the critical glenoid bone loss should be lower than the 20–25% often cited [19, 23, 32, 34]. A case control study published in 2017 showed that an anterior glenoid bone loss of 17.3% or more may result in recurrent shoulder instability after Bankart repair [33]. The amount of glenoid bone loss at which bony procedures are needed to give full stability would, therefore, probably be significantly lower than the 20 to 25% threshold commonly accepted [4, 30]. For this reason, we investigated the sling procedure with a target defect size of 20% anterior glenoid bone loss to ensure instability.

The QTB sling offers better stability compared to 20% anterior glenoid bone loss in all four testing positions. The tendon–bone sling also provides better stability than an intact and ventilated joint in the anterior and anterior-inferior directions in all testing positions, but not in the plain inferior direction. The technique is developed to address anterior instability of the shoulder with bone loss and prevents the motions causing an anterior dislocation of the glenohumeral joint. When performing the QTB sling procedure, all the instrument handling takes place lateral to the conjoined tendon, which potentially reduces the risk of harm to nerves and vessels. The QTB sling may, therefore, be a safe alternative to the Latarjet procedure and other bony transport procedures. The subscapular sling does not alter the anatomy regarding the conjoined tendon, coracoid and nerves [37]; the Latarjet technique may, therefore, serve as a “salvage procedure” in case of failure.

An intact subscapular tendon is crucial to achieve a stabilizing effect. In contrast to the subscapular tenodesis performed in the Putti–Platt procedure [36] and other techniques described recently [7, 24], the subscapular tendon–bone sling achieves a combination of both dynamic and static stabilization without a tenodesis of the subscapular tendon [20] which may prevent over constraint and subsequently, secondary arthrosis. In a previous study of the ST subscapular sling [37], the graft was fixated in 0° of abduction and neutral rotation and a significant reduction in external rotational range of motion at 60° of abduction was observed. To prevent this in the present study; the sling was attached in 30° of abduction and 20° of external rotation of the arm, which assured that no tension was applied to the subscapular tendon. The results of this study reveal no significant reduction in external rotation. The correct tension of the tendon graft sling is difficult to estimate, but by placing the humerus in abduction and external rotation, the biomechanical results show no significant reduction in maximum external rotation. In future clinical testing of the subscapular sling, the proximal tendon graft should, therefore, be attached in the above-described position of the arm.

Blauth et al. [22] proposed the QTB graft for application in anterior cruciate ligament reconstruction in 1984. The QTB graft was chosen in the current study because of its dense bone and the potential to harvest only the superficial part of the tendon, leaving the deep tendon layer intact and thus not compromising the donor site further. Donor site complications may occur, although these have been reported to be minor in most cases [45]. Grafts used for cruciate ligament reconstructions are weakest during the first 12 postoperative weeks, before revascularization and reinforcement begin [8, 21]. A future postoperative rehabilitation protocol may, therefore, include use of a supportive sling and restrictions in load and range of motion.

The 3.75-mm screws are usually used for the Latarjet procedure, and breakage of screws and non-union has been described for this procedure [31]. The incorrect placement of the screws resulted in fracture of the bone block and rupture of the tendon insertion at the tip of the bone block in one specimen. Attaching the bone block with a suture button is potentially a technically easier solution [28]. The angulation of the graft relative to the glenoid is difficult to estimate during arthroscopic surgery. The bone block had a tendency to angulate, causing the block to have a smaller radius than the native glenoid. This does improve the stability of the joint, but in a clinical setting may increase erosion of the humeral cartilage, with subsequent arthritis.

**Fig. 8:**
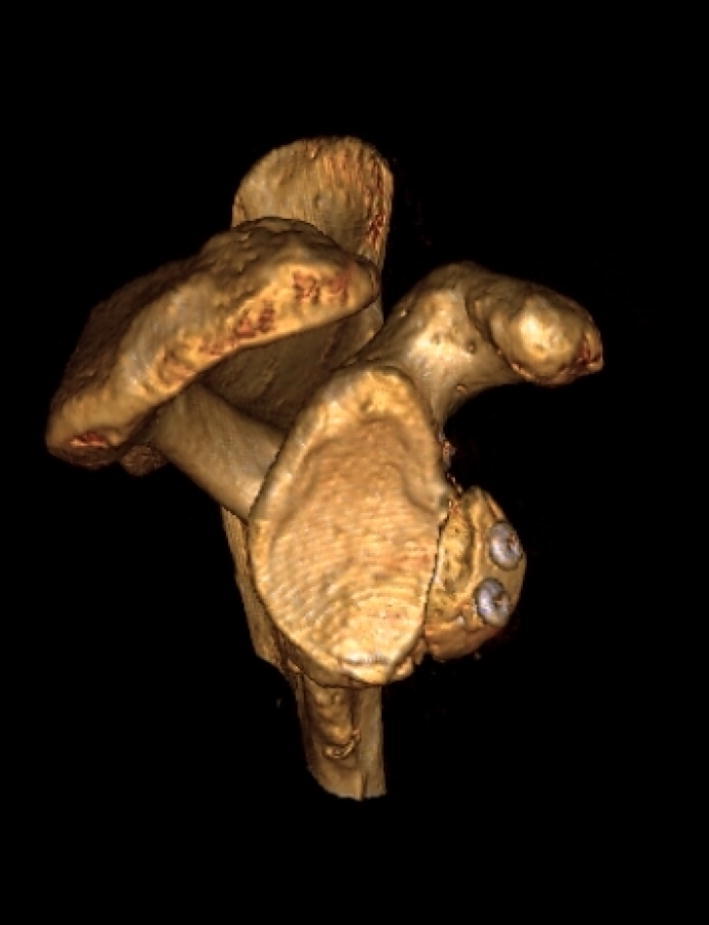
Representative CT image of the bony structures after surgical completion of the sling procedure

The actual width of glenoid bone resection attained was 18% of target which represents 5.1 mm on average; the intended amount was 20% (mean 5.7 mm). Image magnification during arthroscopy may lead to misinterpretation of size and angles [14]; this effect may explain the difference in size of the planned and the executed anterior glenoid bone resection. Postoperative CT scans were used to document the localization of the graft on the glenoid rim and the placement of screws. The cranial end of the bone block was placed during surgery in alignment with the anterior–posterior marking done at 3 o’clock to 9 o’clock. The correct placement of the bone block on the glenoid rim is technically challenging. The CT scans document that the cranial end of all the bone blocks was placed below the 2 o’clock position (Fig. 8), which is higher than the recommended placement of the coracoid bone block in the Latarjet technique [10]. The postoperative CT scans revealed three specimens with rotated bone blocks and screws with a more medial pointing direction than intended. All the specimens were investigated arthroscopically after robot testing prior to refreezing and postoperative CT examination, confirming correct placement and no fracture of the bone block, so we concluded that the rotation of the bone blocks must have occurred during non-intentional rough handling of the cadaveric shoulders before refreezing or while CT scanning.

The following limitations of this study should be considered. The shoulders were not forcibly dislocated anterior inferiorly to imitate a normal anterior shoulder dislocation before the bone lesion was created. Traumatically inducing a lesion may have resulted in a more realistic shoulder joint instability created, but reproducibility of the soft tissue injury and the size of the defect are difficult to attain. We are not aware of any standardized methods to reliably reproduce this injury in cadaver specimens. The study has furthermore not considered the existence of a Hill–Sachs lesion, which is presumably present in a significant number of patients. An engaging Hill–Sachs lesion increases the probability of a second or further dislocation [5, 15, 18, 25]. The increase in the glenoid margins and restrained anterior sliding of the humeral head relative to the glenoid caused by the QTB sling could turn an engaging Hill–Sachs lesion into a non-engaging lesion and thus contribute to better stability. The tendons and muscles surrounding the shoulder joint were only passively loaded during the robot testing. Active muscle loading may have resulted in smaller translations because of the stabilizing function of the rotator cuff. Finally, the results do not represent an in vivo condition which could include tissue scarring, soft tissue healing, bone ingrowth and active motion and adaption of the operated shoulder by the patients. This study is one in a series of biomechanical and clinical studies seeking to develop an alternative arthroscopic surgical option to the existing procedures.

## Conclusion

This experimental study has demonstrated increased stability in the shoulders with the completed subscapular QTB sling. The procedure investigated was performed arthroscopically and does not alter the anatomy with regards to the coracoid, the conjoined tendon or the nerves. Clinical trials must be the next phase before implementing this procedure as a treatment option in patients with anterior shoulder instability and glenoid bone loss.
